# Cortical responses to whole‐body balance perturbations index perturbation magnitude and predict reactive stepping behavior

**DOI:** 10.1111/ejn.14972

**Published:** 2020-09-20

**Authors:** Teodoro Solis‐Escalante, Mitchel Stokkermans, Michael X. Cohen, Vivian Weerdesteyn

**Affiliations:** ^1^ Department of Rehabilitation Donders Institute for Brain, Cognition and Behavior Radboud University Medical Center Nijmegen The Netherlands; ^2^ Department of Neuroinformatics Donders Institute for Brain, Cognition and Behavior Radboud University Nijmegen The Netherlands; ^3^ Sint Maartenskliniek Research Nijmegen The Netherlands

**Keywords:** action monitoring, balance control, electroencephalogram, mobile brain/body imaging, theta rhythm

## Abstract

The goal of this study was to determine whether the cortical responses elicited by whole‐body balance perturbations were similar to established cortical markers of action monitoring. Postural changes imposed by balance perturbations elicit a robust negative potential (N1) and a brisk increase of theta activity in the electroencephalogram recorded over midfrontal scalp areas. Because action monitoring is a cognitive function proposed to detect errors and initiate corrective adjustments, we hypothesized that the possible cortical markers of action monitoring during balance control (N1 potential and theta rhythm) scale with perturbation intensity and the eventual execution of reactive stepping responses (as opposed to feet‐in‐place responses). We recorded high‐density electroencephalogram from eleven young individuals, who participated in an experimental balance assessment. The participants were asked to recover balance following anteroposterior translations of the support surface at various intensities, while attempting to maintain both feet in place. We estimated source‐resolved cortical activity using independent component analysis. Combining time‐frequency decomposition and group‐level general linear modeling of single‐trial responses, we found a significant relation of the interaction between perturbation intensity and stepping responses with multiple cortical features from the midfrontal cortex, including the N1 potential, and theta, alpha, and beta rhythms. Our findings suggest that the cortical responses to balance perturbations index the magnitude of a deviation from a stable postural state to predict the need for reactive stepping responses. We propose that the cortical control of balance may involve cognitive control mechanisms (i.e., action monitoring) that facilitate postural adjustments to maintain postural stability.

## Graphical abstract

This study shows a significant relation between the perturbation‐related N1 potential and the interaction between perturbation intensity with ensuing corrective postural response; which indicates that, for a given perturbation intensity, the magnitude of the N1 potential (and accompanying cortical oscillations) may predict the execution of stepping and feet‐in‐place responses. This provides further evidence that cognitive control mechanisms may regulate reactive postural adjustments.

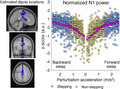

**Abbreviations**
EEG: electroencephalogram
EOG: electrooculogram
ERN: error‐related negativity
ErrP: error‐related potentials
FDR: false discovery rate
FWHM: full‐width at half‐maximum
IC: independent component
ICA: independent component analysis
IIR: infinite impulse response
MNI: Montreal Neurological Institute
PEP: perturbation‐evoked potential
*SD*
: standard deviation
SMA: supplementary motor area

## 1 INTRODUCTION

In everyday activities, we must continuously adjust our posture to maintain balance and avoid falling. The control of human balance and posture requires fast and robust coordination of neural ensembles distributed across multiple levels of the central nervous system (Bolton, 2015; Jacobs & Horak, 2007; Maki & McIlroy, 2007). Although the traditional view is that balance and posture are controlled by the brainstem, basal ganglia, and cerebellum, the cerebral cortex may interact with these structures to maintain balance during goal‐directed movement with varying environmental demands (Jacobs, 2014; Nutt et al., 2011; Takakusaki, 2017).

The cerebral cortex presumably contributes to maintaining postural stability by detecting deviations from a stable postural state and by modulating or initiating appropriate corrective actions, either by adapting the excitability of subcortical postural circuits or by directly contributing to postural responses (Bolton, 2015). The likelihood of cortical contributions to reactive postural responses increases with the latency of the postural response (Jacobs & Horak, 2007). Yet, the cortical responses to external balance perturbations appear in the electroencephalogram (EEG) as early as 30 ms after perturbation onset. Robust cortical responses to balance perturbations appear with broad scalp distribution and rich spectral composition (Peterson & Ferris, 2018; Solis‐Escalante et al., 2019; Varghese et al., 2017) and likely reflect cognitive and sensorimotor processes related to the integration of sensory information associated with sudden postural changes (Dietz et al., 1984; Dietz, Quintern, Berger, et al., 1985), and to the detection of a mismatch between expected and current postural stability (Adkin et al., 2006; Payne, Ting, et al., 2019).

The earliest cortical responses to balance perturbations appear over fronto‐centro‐parietal scalp areas as characteristic event‐related potentials comprising a small positive peak (P1) and a large negative peak (N1), with respective latencies of 30–90 and 90–160 ms relative to perturbation onset (see Varghese et al., 2017 for a comprehensive review). These so‐called perturbation‐evoked potentials (PEP) P1 and N1 are modulated by the physical characteristics (i.e., displacement, velocity, acceleration, and duration) of the balance perturbations. The early P1 potential is thought to represent initial sensory afferences related to proprioception (Dietz et al., 1984; Dietz, Quintern, Berger, 1985) because the P1 potential is suppressed by ischemic deafferentation (Dietz, Quintern, Berger, 1985), suppressed by peripheral nerve stimulation (Staines et al., 2001), and presumably suppressed due to presynaptic inhibition during gait (Dietz et al., 1984; Dietz, Quintern, Berger, 1985; Dietz, Quintern, Berger, et al., 1985). The N1 potential increases with the intensity of the perturbation and its associated destabilizing effect (Dietz et al., 1989; Dietz, Quintern, Berger, et al., 1985; Goel et al., 2018; Mochizuki et al., 2010; Payne, Hajcak, et al., 2019; Staines et al., 2001), which suggests that the N1 potential is at least partially involved in the processing of the multisensory input associated with a sudden change in posture and postural stability. However, the N1 potential is unlikely to represent cortical contributions to early‐phase reactive postural responses as demonstrated by its latency (~150 ms) and its weak correlation with fast reactive muscle responses (Dietz et al., 1989; Dietz, Quintern, Berger, et al., 1985; Mierau et al., 2015; Payne, Hajcak, et al., 2019). Instead, the N1 potential may represent cognitive and sensorimotor processes that modulate late‐phase postural responses (e.g., stepping). Consistent with a possible cognitive function, the N1 potential is strongly affected by psychological factors such as perceived postural threat (Adkin et al., 2008; Mochizuki et al., 2010), the predictability of perturbation characteristics such as onset and intensity (Adkin et al., 2006; Mochizuki et al., 2008, 2009, 2010; Payne, Hajcak, et al., 2019), attention to concurrent tasks (Little & Woollacott, 2015; Quant et al., 2004), and habituation (Mierau et al., 2015; Payne, Hajcak, et al., 2019). For example, imposed changes to postural stability of the same magnitude elicit stronger N1 potentials under conditions of increased postural threat and reduced predictability (Adkin et al., 2008), whereas attention to a concurrent task or repeated exposure to balance perturbations gradually decreases the N1 potential (Mierau et al., 2015). These observations indicate that the N1 potential is internally regulated according to an expected deviation from a current stable posture.

The N1 potential could represent mechanisms of cognitive control (i.e., error detection and action monitoring) for self‐regulation of performance via adaptive behavior. Interestingly, it has been proposed that the N1 potential represents a form of error detection (Adkin et al., 2006; Marlin et al., 2014; Payne, Ting, et al., 2019) because it shares several characteristics with classical error‐related cortical responses. The error‐related negativity (ERN) and the error‐related potentials (ErrP) are cortical responses to the realization of an erroneous action, and have similar latencies and scalp topographies to those of the N1 potential (Chavarriaga et al., 2014; Crowley, 2013). Furthermore, the error‐related responses (ERN/ErrP) scale with the magnitude and consequence of the perceived error and are modulated by prior knowledge about error occurrence (e.g., magnitude, consequence, and timing). This is comparable to how the N1 potential scales with analogous characteristics of a balance perturbation (i.e., perceived postural threat and onset predictability). Direct comparison of the N1 potential elicited by imposed postural changes (low‐intensity balance perturbations) and the ERN/ErrP elicited by erroneous actions (incorrect left/right hand button press during a flanker task) showed that these responses arise from different cortical areas, i.e., the ERN/ErrP originates in the anterior cingulate cortex (ACC), whereas the N1 potential originates from the supplementary motor area (SMA; Marlin et al., 2014). The localization of the N1 potential to the SMA has been repeatedly confirmed (Goel et al., 2018; Mierau et al., 2015; Solis‐Escalante et al., 2019) and interpreted as evidence in favor of a role of the N1 potential in sensorimotor processes (e.g., movement preparation and initiation) over mechanisms of cognitive control (Varghese et al., 2017). Nonetheless, it is important to mention that different aspects of cognitive control (e.g., decision conflict and response error) are associated with activity (including ERN/ErrP) from multiple structures in the posterior midfrontal cortex, including the SMA, pre‐SMA, and the ACC (Bonini et al., 2014; Luu et al., 2000; Ridderinkhof et al., 2004). Therefore, it is possible that the N1 potential and the ERN/ErrP represent different aspects of a general action monitoring system (Payne, Ting, et al., 2019).

Action monitoring refers to the capacity to evaluate the outcome of our actions in order to detect errors and initiate corrective adjustments (Luu et al., 2000; Ridderinkhof et al., 2004). This implies that cortical markers of action monitoring are closely related to adaptive goal‐directed behavior. Indeed, the amplitude of ERN/ErrP correlates with the magnitude of a perceived error and the required corrective response (Debener et al., 2005; Pereira et al., 2017). Similarly, the power of the midfrontal theta rhythm (3–7 Hz) correlates with error detection, response conflict (or uncertainty), and the associated behavioral adaptations (Cavanagh & Frank, 2014; Cohen & Donner, 2013). Besides the ERN/ErrP and the theta rhythm being both correlated with error detection and adaptive behavior, it has been proposed that the ERN/ErrP may be generated through phase resetting of the ongoing theta rhythm (Luu et al., 2004; Trujillo & Allen, 2007; Yeung et al., 2007), suggesting a close interrelation between the ERN/ErrP and the midfrontal theta rhythm. The ERN/ErrP and the midfrontal theta rhythm are considered cortical markers of action monitoring and may be part of a feedback control loop for top‐down regulation of behavior. It remains to be established whether similar mechanisms take part in the control of balance and posture, where the neural activity at cortical levels of the postural control system could reflect action monitoring mechanisms for an internal assessment of postural stability that determines the need for late‐phase balance recovery responses.

In this study we evaluated the association of the cortical responses elicited by balance perturbations with the intensity of the perturbation (as a form of perceived error), the ensuing reactive postural response (as necessary corrective actions), and the interaction between these factors. We were particularly interested in the interaction between perturbation intensity and reactive postural response (stepping vs. non‐stepping) because it underlies a behavioral model of stepping probability and balance capacity, and this behavioral model may mirror the internal processes that regulate postural stability. We hypothesized that the cortical responses to balance perturbations would scale with perturbation intensity and its interaction with the type of postural response, suggesting that the cortical responses to balance perturbations follow the magnitude of the imposed change to postural stability and its associated corrective response. In this way, we investigated whether the cortical responses elicited by whole‐body balance perturbations are consistent with known cortical markers of action monitoring.

We analyzed temporal and spectral parameters of these cortical responses, with special focus on the time period around the N1 potential. We used a wide range of perturbation intensities to investigate the cortical responses elicited by balance perturbations covering the extent of the transition between non‐stepping and stepping responses. This was important because previous studies have been largely limited by the use of small sets of low‐intensity perturbations that exclusively elicit non‐stepping responses (Dietz et al., 1984, 1989; Dietz, Quintern, Berger, et al., 1985; Goel et al., 2018; Mochizuki et al., 2010; Payne, Hajcak, et al., 2019) or by the use of two distinct perturbation intensities (high ‐ low) to elicit stepping and non‐stepping responses (Mochizuki et al., 2010; Omana Moreno, 2017). Furthermore, we analyzed a wide range of spectral components to better understand the modulations of cortical rhythms with respect to the perturbation intensity and reactive responses. We anticipated that the power of the theta rhythm would be modulated by perturbation intensity and reactive responses, due to the known role of the midfrontal theta rhythm as cortical marker of action monitoring, but also because transient conditions of reduced postural stability (caused by external perturbations or natural sway) elicit a brief power increase of the theta rhythm in fronto‐centro‐parietal scalp areas (Peterson & Ferris, 2018; Slobounov et al., 2009; Solis‐Escalante et al., 2019) and because the power of the theta rhythm covaries with postural demand (Hülsdünker et al., 2015; Mierau et al., 2017). Other spectral features were analyzed because perturbations to standing balance elicit a broadband power increase of frequencies between 3–17 Hz within 500 ms from the perturbation onset (Peterson & Ferris, 2018; Solis‐Escalante et al., 2019; Varghese et al., 2014); yet, their association with perturbation intensity remains largely unexplored. Our analysis offered the possibility to identify specific cortical rhythms that may be associated with distinct cognitive and motor functions. An association of temporal or spectral parameters of the cortical responses with perturbation intensity and reactive postural responses would provide further evidence about the neural correlates of top‐down regulation of reactive postural responses.

## 2 MATERIALS AND METHODS

### 2.1 Participants

Eleven young able‐body individuals participated in this study (age: 26 ± 3 years old, four female). None of the participants had self‐reported history of neurological or neuromuscular disease or any other impairments that limited their involvement in the experiment. The experiments were undertaken with the understanding and written consent of each participant. The study protocol was approved by the Research Ethics Committee of the Radboud University Medical Center (Nijmegen, The Netherlands; Dossier 2018‐4970). The experiments were conducted in accordance with the Declaration of Helsinki.

### 2.2 Experimental paradigm

**FIGURE 1:**
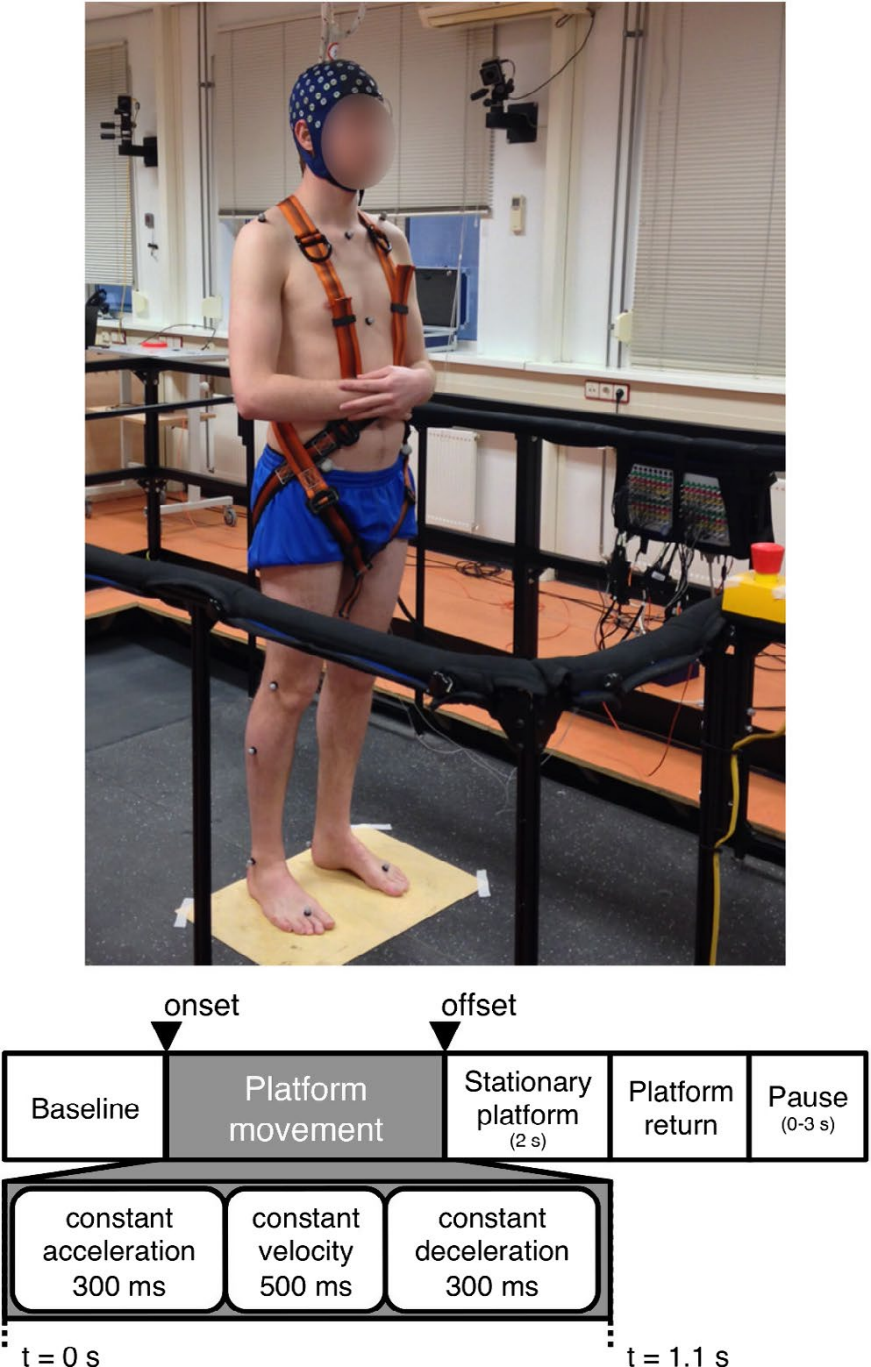
Experimental setup and trial timing. Top: The participants stood in the middle of a movable platform with arms crossed and feet placed apart at shoulder width. Bottom: The balance perturbations were ramp‐and‐hold platform translations consisting of three phases: constant acceleration, constant velocity, and constant deceleration. At the end of the displacement the platform remained stationary for 2 s

The experiments were conducted with the Radboud Falls Simulator, a dynamic posturography system for investigating standing balance (Nonnekes et al., 2013). During the experiments, the participants stood in the middle of a movable platform with arms crossed and feet placed apart at shoulder width. Figure 1 illustrates the experimental setup and the trial timing.

The participants were instructed to maintain standing balance by keeping both feet in place, in response to sudden balance perturbations. The balance perturbations were ramp‐and‐hold translations of the movable platform consisting of three phases: constant acceleration (300 ms), constant velocity (500 ms), and constant deceleration (300 ms). At the end of the displacement the platform remained stationary for 2 s before gently returning to its initial position. The intensity of the perturbations was controlled by varying the acceleration of the translations from 0.125 to 2.5 m/s^2^ (increments of 0.125 m/s^2^, leading to 20 accelerations). The direction of the translation was either forward or backward, and thus there were a total of 40 different perturbations (20 accelerations × 2 directions). The higher perturbation intensities required the execution of reactive stepping to maintain standing balance. Forward translation of the platform elicited postural sway and an eventual step in the backward direction; similarly, backward translation of the platform elicited postural sway and an eventual step in the forward direction. Henceforth, we refer to the direction of the postural sway and eventual stepping response, unless specifically indicated as the direction of the platform translation. Participants were made aware of this and they were assured that stepping could not be avoided for a fair amount of perturbation intensities. Nonetheless, participants were encouraged to keep both feet in place throughout the experiment.

The perturbations were arranged into blocks of 10 forward and 10 backward translations with intensities uniformly distributed across the range of accelerations (same intensities in both directions per block). The order of the perturbations was randomized within each block and across participants. The inter‐trial interval randomly varied between 3 and 5 s (with uniform distribution). Depending on the duration of preparation time and resting breaks, participants completed 120 or 160 experimental trials in one experimental session. Due to the arrangement of the perturbation blocks, each distinct perturbation was tested three to four times. Importantly, the participants could not predict timing onset, direction, or intensity of the perturbation.

To prevent fatigue, short pauses lasting 3–5 min were encouraged between blocks. Prior to the experiment, participants practiced with one block of perturbations to familiarize themselves with the task. The familiarization trials were not included in the analysis.

### 2.3 Data collection

We recorded high‐density EEG using an electrode cap with 126 Ag‐AgCl electrodes (WaveGuard, ANT Neuro, The Netherlands). The electrodes were distributed across the scalp according to the five percent electrode system (Oostenveld & Praamstra, 2001). The ground electrode was placed on the left mastoid using an adhesive Ag‐AgCl electrode. In addition, two‐channel electrooculogram (EOG) was recorded using adhesive Ag‐AgCl electrodes placed slightly above the nasion and at the outer canthus of the left eye. The ground electrode was used for both the EEG and the EOG channels. A biosignal amplifier (REFA System, TMSi, The Netherlands) recorded the EEG/EOG at 2048 Hz without any filters, except for a built‐in antialiasing low‐pass filter. The 128 signals (i.e., EEG and EOG) were referenced to the common average during acquisition. Ground reaction forces were recorded from two force plates (AMTI custom 6 axis composite force platform, Watertown, MA, USA; size: 60 × 180 cm each; sampling rate: 2,000 Hz) embedded in the movable platform. Each force plate recorded ground reaction forces from one foot. Synchronization triggers indicating the onset and offset of the platform movement were generated by the platform controller and simultaneously recorded with the EEG/EOG signals and the ground reaction forces.

Before beginning the experiment, EEG (and EOG) signals were recorded for a set of control conditions during quiet stance. These control conditions were short recordings (approx. one minute each) involving overt eye movement (blinking, lateral movement, eye rolling), head/neck movement (rotation, flexion/extension, lateral flexion), facial expressions (movements of mouth, lips, nose, and eyebrows), and jaw clenching; with an additional one minute of quiet stance with eyes open. These recordings were intended to assist the separation of sources of physiological noise by providing clear examples of their source activity that could be modeled as independent sources.

### 2.4 Detection of reactive stepping responses

The ground reaction forces were exported to C3D format and later imported into MATLAB (The Mathworks, Inc.) for analysis. Reactive stepping responses were detected from the vertical component of the ground reaction forces using threshold detection. The vertical component of the ground reaction forces measures the force applied to each side of the movable platform, corresponding with each leg. During quiet stance the sum of the left and right ground reaction forces equals the bodyweight of the participants (mass in kg) multiplied by the acceleration of gravity on Earth (~9.8 m/s^2^). The vertical force components from each force plate were low‐pass filtered at 20 Hz (5th order Butterworth IIR filters, zero‐phase shift) and compared against a threshold of 10 N (~1 kg). Values below this threshold indicate that one of the feet has been lifted from one of the force plates. Reactive stepping responses were detected if they occurred within 1 s from perturbation onset; otherwise, the response was classified as non‐stepping (feet‐in‐place). Participants were allowed to step with either leg.

### 2.5 EEG analysis

#### 2.5.1 Preprocessing

The EEG was analyzed with MATLAB using custom scripts and incorporating functions from EEGLAB (Delorme & Makeig, 2004). The EEG was filtered between 1 and 200 Hz (consecutive high‐pass and low‐pass 5th order Butterworth IIR filters, zero‐phase shift) and downsampled to 512 Hz. The EEG and EOG recordings from control conditions and experimental blocks were concatenated. Highly contaminated channels were identified by visual inspection and removed from the recordings. On average, 126 channels remained for analysis (*SD* ± 1.7). The remaining channels were re‐referenced to the common average. The data were visually inspected for segments with cable movements or electrode disconnection, which were removed from the data.

#### 2.5.2 Estimation of source‐resolved activity

Independent component analysis (ICA) was used to estimate source‐resolved brain activity from the high‐density EEG (Gramann et al., 2014; Makeig et al., 2009) and to reduce the influence of other sources of physiological noise (e.g., electromyogram and electrocardiogram; Gwin et al., 2010; Kline et al., 2015; Oliveira et al., 2016; Snyder et al., 2015). This approach is in line with previous studies on cortical dynamics during whole‐body movement and balance control (Gwin et al., 2011; Peterson & Ferris, 2018; Sipp et al., 2013; Solis‐Escalante et al., 2019; Varghese et al., 2014). Because the EEG was referenced to the common average, a principal component analysis was used before ICA simply to remove the principal component with the lowest eigenvalue (null‐space; Artoni et al., 2018).

Following the ICA, the source‐resolved activity was segmented into epochs from −2 to 9 s relative to perturbation onset. Per participant, one independent component (IC) was identified as the likely source of the N1 potential by inspection of the event‐related potential associated with each IC. All candidate ICs were further evaluated as likely brain sources based on the residual variance of an equivalent current dipole fitted to their scalp projections. The equivalent current dipoles were fitted using a four‐shell spherical head model and standard electrode positions (DIPFIT toolbox within EEGLAB, Oostenveld & Oostendorp, 2002). The equivalent current dipoles provide an estimation (limited in spatial resolution) of the likely location of the source‐resolved N1, which assists the validation of a dipolar topography of the scalp projection and a physiologically plausible location (Kline et al., 2015; Snyder et al., 2015). Thus the objective of the source localization analysis was to provide additional information on the distribution of individual IC scalp maps, to answer the questions whether the scalp map has a dipolar distribution (indicated by its residual variance) and whether the scalp map is likely to represent a cortical source (indicated by its spatial location).

The location of the equivalent current dipoles calculated by the DIPFIT toolbox are given in Talairach coordinates. The corresponding Broadmann areas were found using the online application mni2tal (available at https://bioimagesuiteweb.github.io/webapp/mni2tal.html) from the Yale BioImage Suite Package (Lacadie et al., 2008).

#### 2.5.3 Time and time‐frequency domain cortical parameters

The signal of the estimated source‐resolved N1 potential was analyzed to quantify single‐trial amplitude and latency. A copy of the source‐resolved signal was low‐pass filtered at 30 Hz (5th order Butterworth IIR filter, zero‐phase shift) and the single‐trial amplitude and latency were identified as the largest negative peak within 300 ms from perturbation onset. The single‐trial amplitude and latency were stored for analysis.

To quantify spectral parameters, the estimated source‐resolved signal was analyzed in the time‐frequency domain by convolving this signal with a set of complex Morlet wavelets, defined as complex sine waves tapered by a Gaussian (Cohen, 2019). The frequencies of the wavelets ranged from 2 to 50 Hz in 30 steps (logarithmically spaced). The full‐width at half‐maximum (FWHM) ranged from 800 to 200 ms, decreasing with increasing wavelet peak frequency. This corresponded to a spectral FWHM range of 1.7–7.2 Hz.

Event‐related parameters were extracted from the single‐trial source‐resolved signal in time domain and the time‐frequency domain for 101 time points within ±500 ms (time resolution: 10 ms), relative to the trial‐specific N1 latency. Thus, there were 101 time domain parameters and 101 × 30 time‐frequency domain parameters. All parameters were transformed to logarithmic power (10 log_10_ (|parameter*_x_* |^2^)), for consistency in analyses and interpretation of temporal and spectral features.

### 2.6 Trial rejection

After selection of the N1 component, the source‐resolved activity was visually inspected once again for possible artifacts (e.g., movement artifacts or excessive contamination from muscular activity) within ±2 s from perturbation onset. Then, single‐trials with N1 amplitudes or latencies beyond ±3 *SD* from the mean were rejected. Trial rejection was separately conducted for each participant. The remaining trials were time‐locked to the N1 latency and visually inspected in the interval −1 to 1.5 s (relative to N1 latency). On average, there were 125 trials (*SD* ± 24) per participant.

### 2.7 Event‐related potentials and spectral modulations

For the purpose of visualization, grand average event‐related potentials associated with stepping and non‐stepping responses were computed in the forward and backward direction. The source‐resolved signals were normalized on a trial‐by‐trial basis (z‐score across time points), time‐locked to the N1 latency, and averaged across trials from the same condition.

Similarly, the grand average event‐related spectral modulations were computed per condition. Single‐trial spectrograms were computed following the time‐frequency analysis described in the previous section (i.e., convolution with complex Morlet wavelets). The spectrograms were transformed to logarithmic power and a trial‐specific baseline was computed as the mean (log transformed) spectrum from the interval −1.5 to −0.5 s, relative to perturbation onset. The baseline was subtracted from its corresponding trial and the baseline‐corrected spectrograms were time‐locked to the N1 latency. Finally, time‐frequency maps showing the mean event‐related spectral modulations (i.e., power changes relative to baseline) were computed by averaging the spectrograms across trials from the same condition. The statistical significance of the spectral modulations was estimated for each time‐frequency bin from its 95% confidence interval (bootstrap, *n* = 200).

### 2.8 Relation of cortical parameters with perturbation intensity and stepping behavior

The effects of perturbation intensity and stepping behavior on the cortical parameters were analyzed with the model for general linear regression:

$$C_{x}∼\beta_{0}+\beta_{1}\cdot \text{ACCEL}+\beta_{2}\cdot \text{STEP}+\beta_{3}\cdot \text{ACCEL}\times \text{STEP,}$$

where the regression coefficients β_1_ and β_2_ indicate the main effects of the perturbation intensity (i.e., acceleration: ACCEL) and the dummy‐coded stepping behavior (STEP), respectively; and the regression coefficient β_3_ indicates the effect of their interaction (ACCEL × STEP). The null‐hypothesis that there is no significant relation with the cortical parameters C*_x_* corresponds to regression coefficients equal to zero. The null‐hypothesis can be rejected if the confidence interval of a given regression coefficient does not include zero.

Regression analyses were conducted at group‐level using pooled trials from all participants, after participant‐specific normalization (z‐score across trials) of the cortical parameters. The analyses were performed separately for forward and backward stepping directions, with time and time‐frequency parameters. An additional regression analysis was conducted to determine the effects of perturbation intensity and stepping behavior on the N1 latency (after participant‐specific normalization).

The significance of the regression analysis was evaluated with an *F* test and the significance of the regression coefficients with a *t* test. Statistical significance was assessed for critical α = 0.01. Given the multiple regression analyses computed in time and time‐frequency domains, *p*‐values were corrected for false discovery rate (FDR; Benjamini & Yekutieli, 2001).

## 3 RESULTS

### 3.1 Reactive stepping responses (behavioral analysis)

**FIGURE 2:**
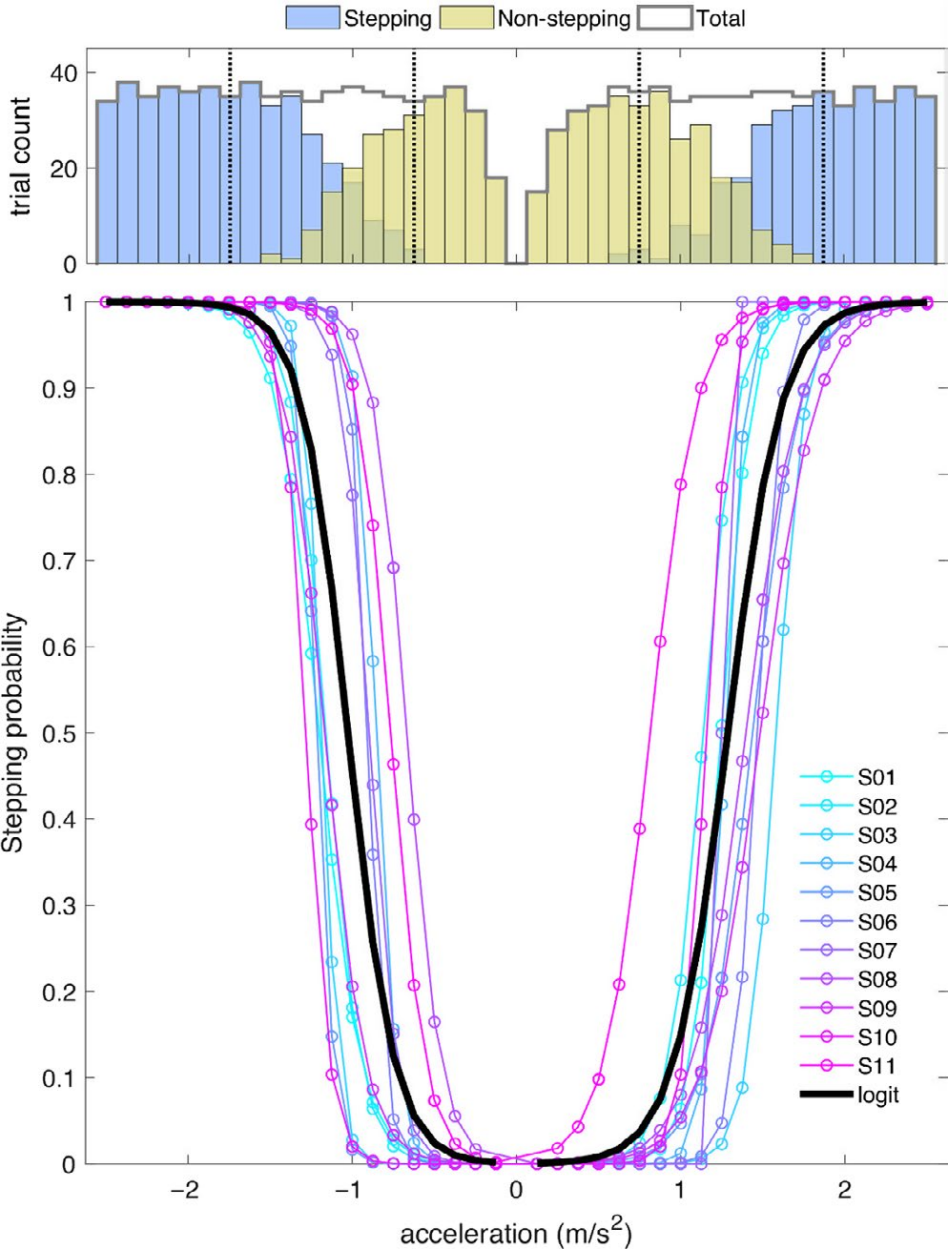
Trial distribution and stepping probabilities (group‐level). Top: Total trial count per acceleration and distribution of stepping (blue) and non‐stepping (yellow) trials. The distribution of the total number of trials was near‐uniform in the backward stepping (negative accelerations) and forward stepping (positive accelerations) direction. Consistent with anatomical and functional constraints, the proportion of stepping trials was higher in the backward direction (see text). Bottom: The stepping probability as a function of perturbation intensity was computed for each direction using logistic regression. Individual probability curves are shown in cold colors (blue to magenta) and the group‐level probability is shown with a thick black line

Table 1 presents the total number of trials per condition and the mean latencies of the reactive stepping responses (foot‐off detection). Importantly, the forward and backward directions refer to the direction of postural sway and eventual stepping. The conditions were defined on basis of postural sway direction (forward vs. backward) and the ensuing reactive response (stepping vs. non‐stepping), irrespective of the perturbation intensity. These latencies are relative to perturbation onset and averaged across stepping responses at varying perturbation intensities. Figure 2 shows the distribution of trials over perturbation intensities (i.e., accelerations) and the estimated stepping probability (computed via logistic regression).

**TABLE 1:** Number of trials and average stepping response latency (ms) per condition

	Backward			Forward		
	Stepping		Non‐stepping	Stepping		Non‐stepping
	Trials	Foot‐off latency	Trials	Trials	Foot‐off latency	Trials
S01	25	411.7	17	20	572.5	15
S02	31	598.5	23	31	632.5	22
S03	35	600.9	24	24	745.9	35
S04	41	318.2	18	31	387.8	27
S05	43	472.2	36	36	688.7	36
S06	51	448.5	22	35	607.7	38
S07	40	443.6	16	31	468.6	25
S08	45	306.3	9	27	386.7	31
S09	33	371.1	30	27	540.6	36
S10	41	499.2	37	45	450.4	31
S11	57	287.2	21	54	397.9	19
Pooled	442	422.4	253	361	521.8	315
*SD* (pooled)		170.1			172.8	

There were 695 trials challenging postural stability in the backward direction and 676 trials challenging postural stability in the forward direction. The mean number of trials per participant was not significantly different between the two directions (two‐tailed paired *t* test; *t*(10) = 1.71, *p* = .118) and the distribution of trials over intensities was close to uniform (Kolmogorov–Smirnov tests for uniform distributions between 0.125 and 2.500 m/s^2^; backward: *D*(695) = 0.051, *p* = .052; forward: *D*(676) = 0.054, *p* = .038; see Figure 2). In both directions, more than half of the trials elicited reactive stepping responses (backward: 63.3%, forward: 53.4%), but the proportion of stepping trials was significantly larger for the backward direction (chi‐squared test; χ^2^ = 14.68, *p* = 1.27e−04). Consistent with these observations, the stepping probability models estimate that 50% stepping probability (at group level) corresponds to 1.02 m/s^2^ for the backward stepping and 1.28 m/s^2^ for the forward stepping direction; with the limits for 25% and 75% stepping probability at [0.87, 1.18] m/s^2^ and [1.10, 1.46] m/s^2^ respectively. The pooled data showed only non‐stepping responses for perturbation intensities smaller or equal to 0.5 m/s^2^ in both directions. Similarly, stepping responses for perturbation intensities greater than 1.5 m/s^2^ for backward stepping and 1.75 m/s^2^ for forward stepping (see histogram in Figure 2). The mean stepping latency was significantly shorter for the backward stepping direction (two‐tailed paired *t* test; *t*(10) = −4.33, *p* = .001).

### 3.2 Visualization of event‐related cortical responses

**FIGURE 3:**
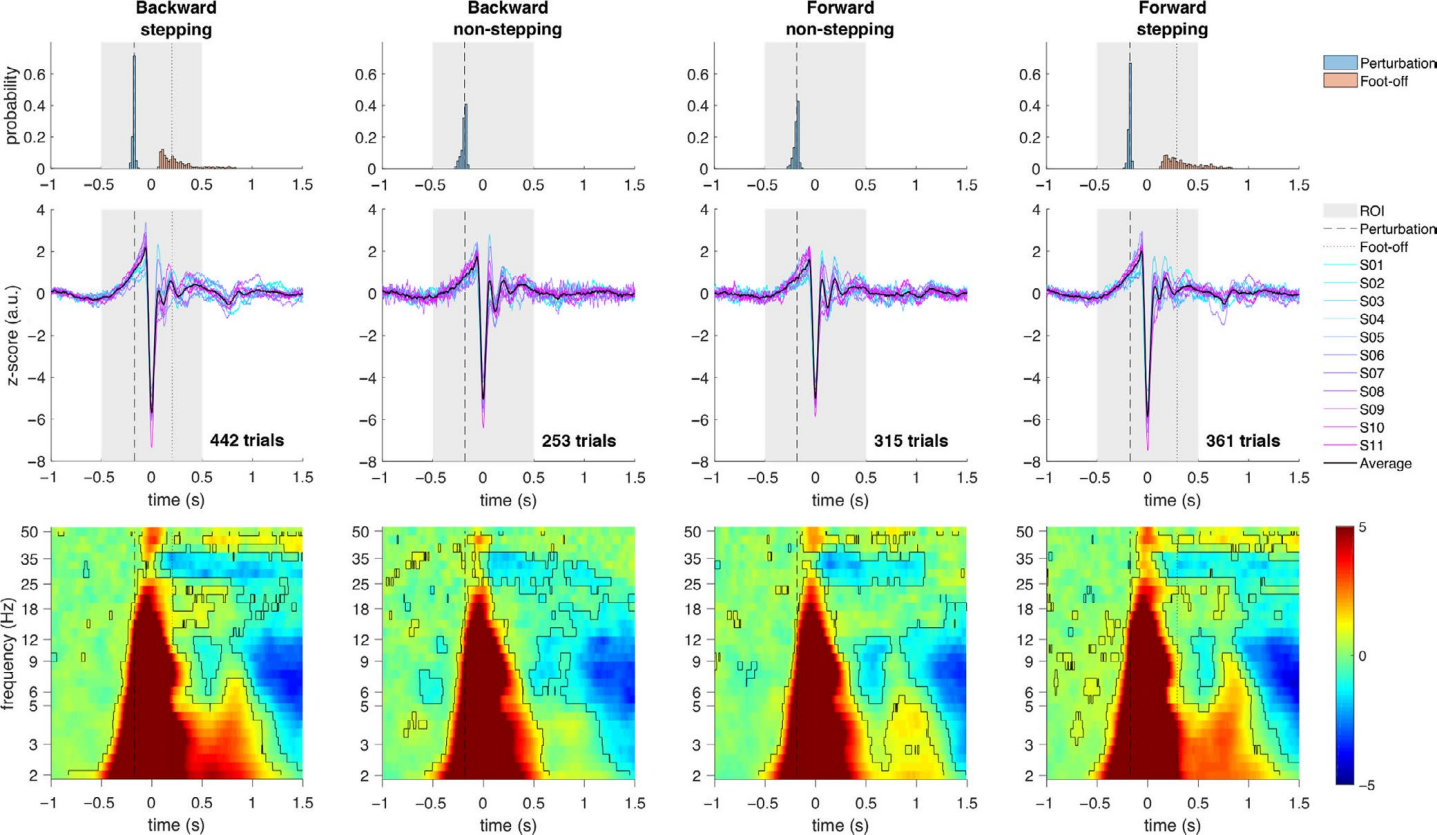
Event‐related potentials and event‐related spectral modulations. Characteristic cortical responses to balance perturbations in time domain (middle row) and time‐frequency domain (bottom row), shown relative to the peak amplitude of the N1 potential (*t* = 0 s) and in comparison with the distribution (top row) of perturbation onset (blue) and foot‐off onset (pink) latencies. The vertical dashed line indicates the median latency of the perturbation onset (backward: stepping −171.9 ms, non‐stepping −183.6 ms; forward: stepping −173.8 ms, non‐stepping −183.6 ms) and the vertical dotted line indicates the median foot‐off latency (backward: 202.6 ms; forward: 289.9 ms). The region of interest (ROI, shown with a gray band) extended from −500 to 500 ms relative to the N1 potential, including the period between median perturbation onset and foot‐off latencies. The time domain plots show a typical N1 potential for each individual (blue to magenta) and the group‐level average (thick black line). The time‐frequency domain maps show the grouplevel average power modulations, with typical broadband power increase (warm colors). The black contour shows power increase/decrease significantly different from baseline (*p* < .05)

In Figure 3, the event‐related potentials show the strong negative peak of the N1 potential (*t* = 0 s) and the characteristics of the cortical response to balance perturbations (Varghese et al., 2017); namely, a slow potential shift preceding perturbation onset, followed by P1 (positive) and N1 (negative) potentials and late potentials of varying latency and amplitude approximately within 400 ms after perturbation onset. The event‐related spectral modulations show a broadband power increase over the frequencies of the theta, alpha, beta, and gamma rhythms, occurring shortly after perturbation onset and coinciding with the N1 potential. In general, the initial broadband power increase is followed by power decrease over the frequencies of the alpha and low‐gamma rhythms. This spectral modulation pattern is characteristic of cortical responses to balance perturbations (Peterson & Ferris, 2018; Solis‐Escalante et al., 2019; Varghese et al., 2017). The visualization of event‐related cortical responses is meant to provide an overview of the time and time‐domain characteristics of the conditions defined on basis of postural sway direction (forward vs. backward) and the ensuing reactive response (stepping vs. non‐stepping), irrespective of the perturbation intensity.

### 3.3 Mean N1 latency

Table 2 presents the mean N1 latency (relative to perturbation onset) for each participant and for the pooled data. Noteworthy, the mean latency per condition was obtained by averaging across trials within the conditions defined on basis of postural sway direction (forward vs. backward) and the ensuing reactive response (stepping vs. non‐stepping), irrespective of the perturbation intensity. Within each direction, the mean N1 latency was significantly shorter for stepping responses than for non‐stepping responses (two‐tailed paired *t* tests; backward: *t*(10) = −13.3, *p* = 1.08e−07; forward: *t*(10) = −7.05, *p* = 3.50e−05). The mean N1 potential latency (cortical response) preceded the mean stepping response latency (behavioral response) in either direction (two‐tailed paired *t* test; backward: *t*(10) = −8.05, *p* = 1.11e−05; forward: *t*(10) = −9.60, *p* = 2.29e−06; average mean latency difference backward stepping: 257 ms, range: 117–433 ms; average mean latency difference forward stepping: 358 ms, range: 212–569 ms). Histograms of the stepping response latency, relative to the N1 potential, are shown in Figure 3 (previous section), together with the average event‐related potentials and event‐related spectral modulations per condition.

**TABLE 2:** Average N1 latency (ms) per condition

	Backward		Forward	
	Stepping	Non‐stepping	Stepping	Non‐stepping
S01	179.0	195.4	176.4	190.4
S02	189.9	206.2	193.2	203.8
S03	167.9	187.9	176.3	184.4
S04	174.1	183.5	175.7	182.0
S05	177.3	192.1	180.6	187.2
S06	168.4	177.9	170.0	175.1
S07	178.0	196.4	177.9	196.4
S08	176.4	190.3	173.3	188.7
S09	168.4	185.0	167.1	177.4
S10	175.8	188.7	173.2	193.4
S11	170.2	191.4	173.8	195.2
Pooled	174.5	190.2	176.0	187.1
*SD* (pooled)	11.7	23.5	11.8	23.1

### 3.4 Estimated location of the N1 cortical source

**FIGURE 4:**
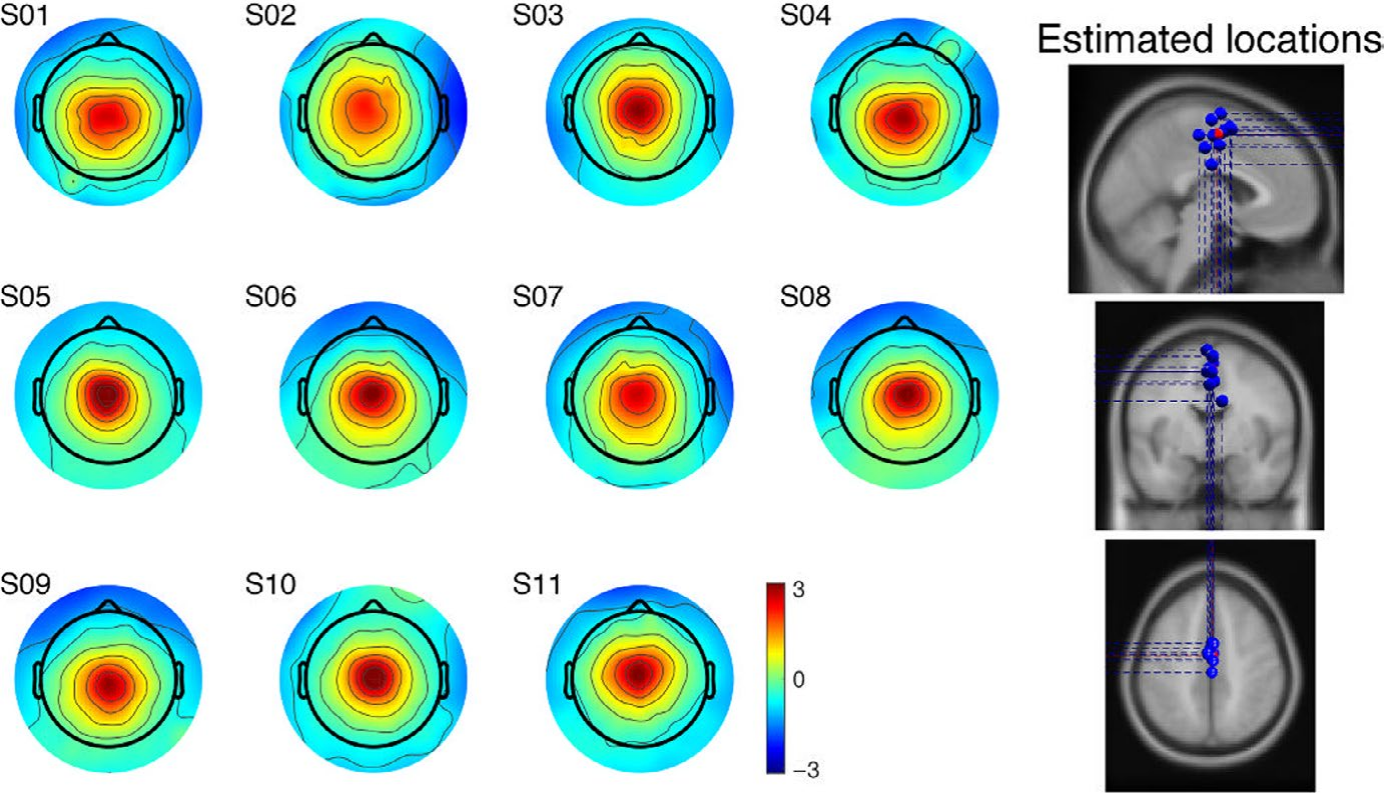
Individual IC scalp maps and estimated cortical source locations. The IC scalp maps of each participant are qualitatively similar and suggest a dipolar topography consistent with the residual variance in Table 3. The similarities are also shown in the estimated cortical location of the equivalent current dipoles (blue, individual participants; red, cluster centroid)

Table 3 lists the estimated location and residual variance of the equivalent current dipoles associated with the ICs identified as the likely cortical sources of the N1 potential. The locations consistently indicated a cortical source in the posterior midline frontal cortex, with Broadmann area 6 (premotor cortex/supplementary motor area) as the most common location. The average residual variance was 2.65% (*SD* ± 0.90%). Figure 4 shows the topography of the scalp projections (i.e., the IC scalp map) and the locations of the equivalent current dipoles.

**TABLE 3:** Estimated cortical source location

	Talairach coordinates			Residual variance (%)	Location and Brodmann area
	*X*	*Y*	*Z*		
S01	0	−16	40	2.23	Posterior cingulate L
					Left BA24
S02	9	−12	28	4.27	Posterior cingulate R
					—
S03	−2	−6	49	3.19	Paracentral L
					Left BA6
S04	1	−21	49	4.03	Paracentral R
					Right BA6
S05	−2	−3	64	1.25	Paracentral L
					—
S06	1	4	53	2.02	Superior frontal R
					Right BA6
S07	3	−5	42	2.44	Posterior cingulate R
					Right BA32
S08	3	3	54	3.05	Superior frontal R
					Right BA6
S09	1	−10	49	2.13	Paracentral R
					Right BA6
S10	2	−10	60	2.18	Paracentral R
					Right BA6
S11	0	−1	52	2.30	Superior frontal L
					Left BA6
Centroid	2	−7	49	—	Right BA6

### 3.5 Relation of cortical parameters with perturbation intensity and stepping behavior

#### 3.5.1 Single‐trial N1 characteristics

**FIGURE 5:**
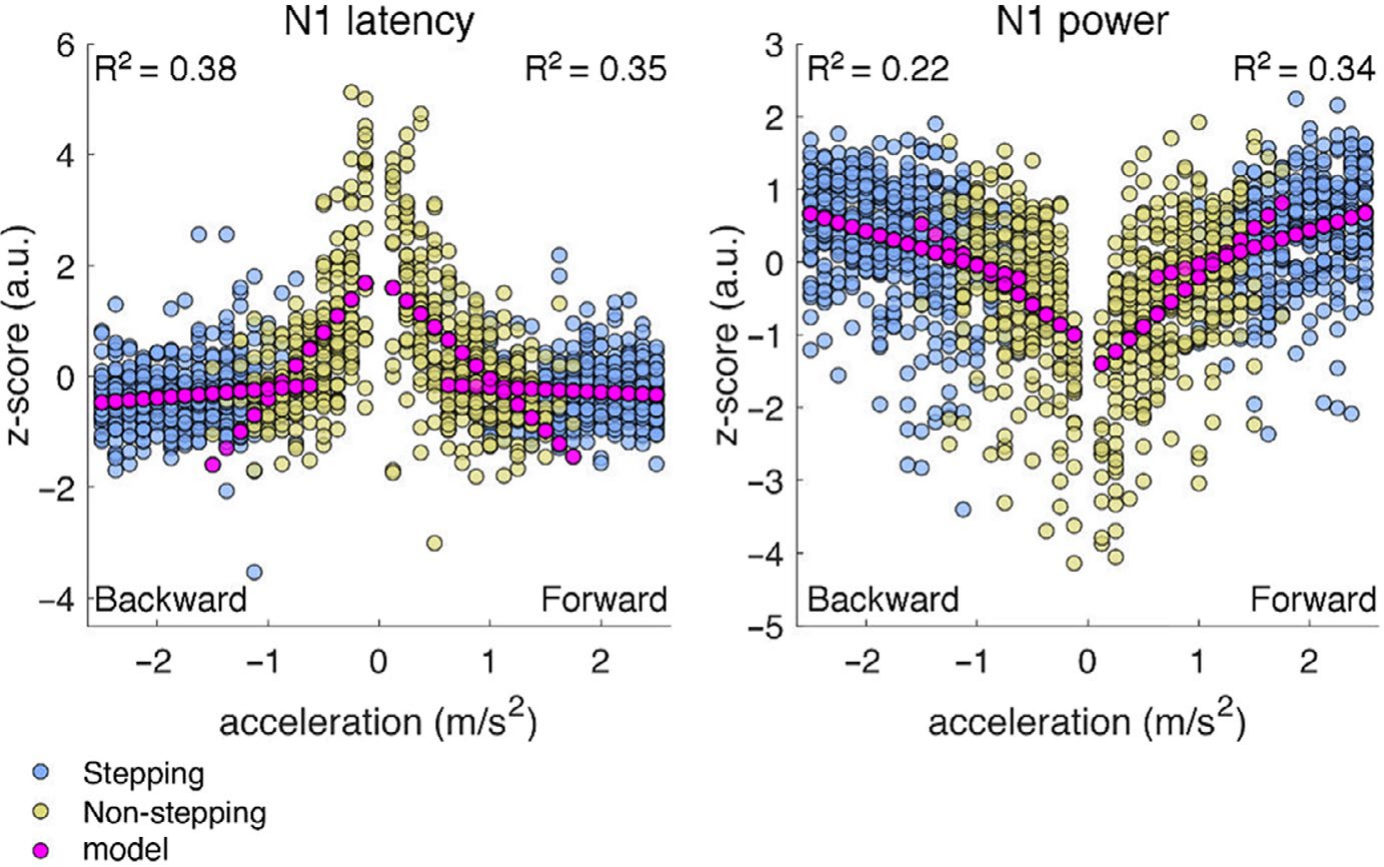
Relation of perturbation intensity and stepping responses with N1 characteristics. Distribution of the pooled single‐trial latency (left) and power of the peak N1 amplitude (right). The data of each participant was normalized by computing the z‐score across trials (including all perturbation intensities and directions). In a previous step, the peak N1 amplitude was transformed to logarithmic power for comparison with the analyses on spectral parameters. The x‐axis indicates perturbation intensity (i.e., acceleration magnitude) multiplied by the sign of the sway direction (negative: backward sway; positive: forward sway). Blue circles indicate trials with a stepping response and yellow circles indicate trials with a non‐stepping response. The corresponding regression models are shown with magenta circles. The determination (*R* ^2^) and regression coefficients (β, not shown) are significant (*p* < .01). The relation with stepping behavior is indicated by the change in slope of the regression models seen between trials with stepping and nonstepping responses

Figure 5 shows the pooled single‐trial latency and power of the peak N1 amplitude together with the corresponding regression models. All determination and regression coefficients indicate a significant (*p* < .01) relation of perturbation intensity, stepping behavior, and the interaction between these factors with the characteristics of the N1 potential. In general, for perturbations that challenge postural stability in either direction, the latency and power of the peak N1 rapidly change with perturbation intensity in trials with non‐stepping responses. The scaling with perturbation intensity is attenuated in trials with stepping responses, which is indicative of the interaction effect.

The distribution of the pooled data and the corresponding regression models for the power of the peak N1 amplitude (Figure 5) are representative of the temporal and spectral parameters (see Figure S1). Overall, the temporal and spectral cortical parameters analyzed here scale with perturbation intensity and this scaling is attenuated from non‐stepping to stepping responses. The cortical parameters related to stepping responses have larger magnitudes and are less affected by perturbation intensity than the cortical parameters related to non‐stepping responses.

#### 3.5.2 Time domain parameters

**FIGURE 6:**
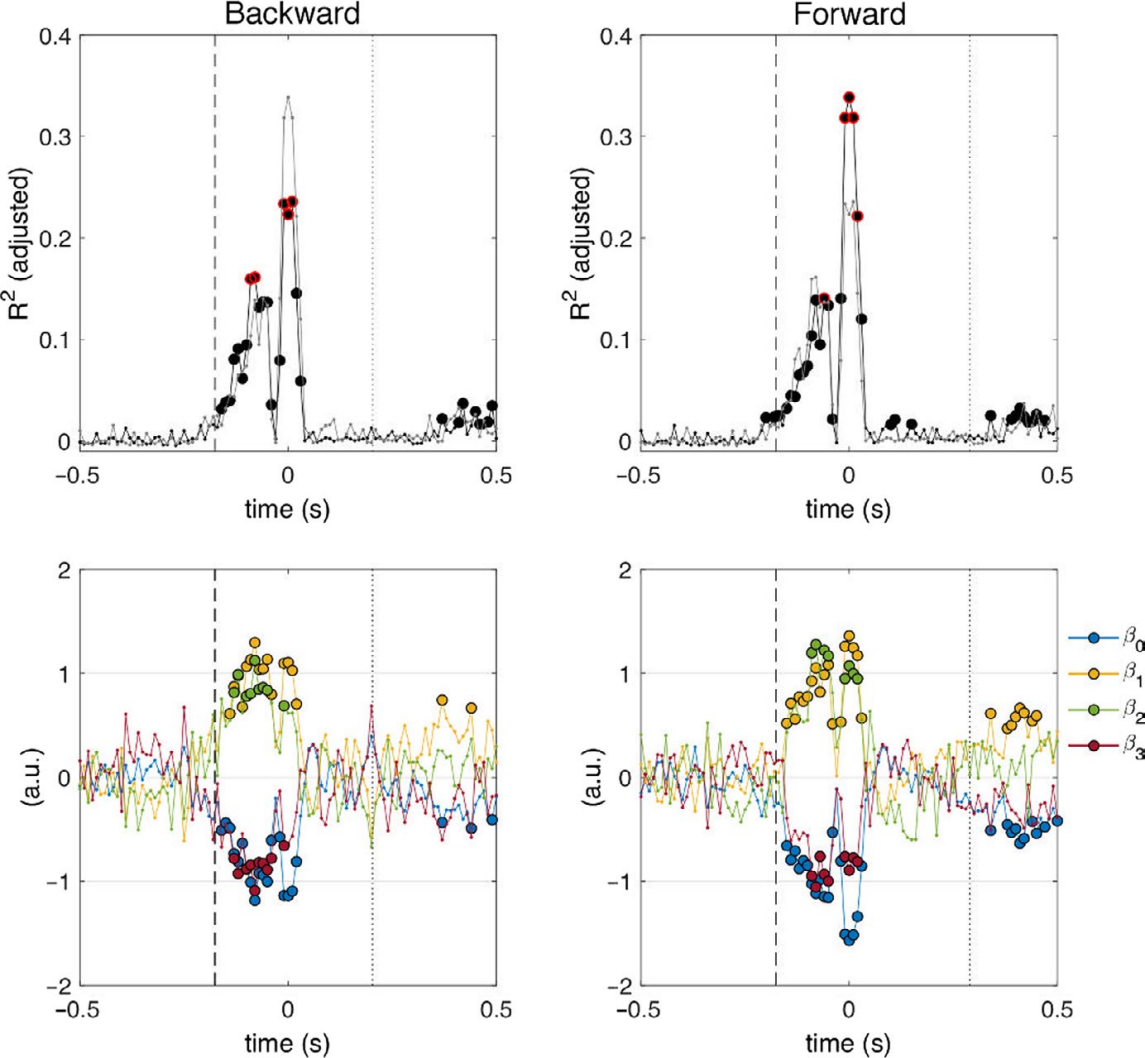
Relation of perturbation intensity and stepping responses with time domain parameters. Timedependent adjusted determination coefficient (top) and regression coefficients (bottom) shown relative to the peak amplitude of the N1 potential (*t* = 0 s). The regression coefficient β_0_ is the intercept of the model, β_1_ indicates the effect of acceleration, β_2_ indicates the effect of distinct reactive postural responses, and β_3_ indicates the effect of the interaction between acceleration and distinct reactive postural responses. The vertical dashed line indicates the median perturbation onset latency (backward and forward: −175.8 ms) and the vertical dotted line indicates the median foot‐off latency (backward: 202.6 ms; forward: 289.9 ms) Filled circles indicate significant coefficient values (*p* < .01, FDR corrected). The top 5% determination coefficients (per direction, across all time points) are indicated with a red edge (top row only). For comparison, the time‐dependent determination coefficient for the opposite direction is overlaid with a gray thin line

Figure 6 shows the adjusted determination coefficient, together with the corresponding regression coefficients, obtained from the regression analyses using time domain parameters. The coefficients of the regression model indicate: β_0_ the intercept, β_1_ the effect of acceleration, β_2_ the effect of distinct reactive postural responses (dummy values: non‐stepping = 0 and stepping = 1), and β_3_ the effect of the interaction between acceleration and distinct reactive postural responses. The regression coefficients show a statistically significant effect of perturbation intensity from −140 to 30 ms (relative to the N1 potential), for perturbations that challenge postural stability in the backward (*p* < .0012) and forward (*p* < .0026) directions. A statistically significant effect of stepping behavior is shown from −130 to −50 ms for backward direction (*p* < .0007); and from −90 to −50 ms and from −10 to 20 ms for forward direction (*p* < .0002). The interaction between perturbation intensity and stepping behavior is also statistically significant in these intervals (backward: *p* < .0010; forward: *p* < .0007). These results show a relation of the N1 potential with perturbation intensity and stepping behavior, as well as their interaction.

#### 3.5.3 Time‐frequency domain parameters

**FIGURE 7:**
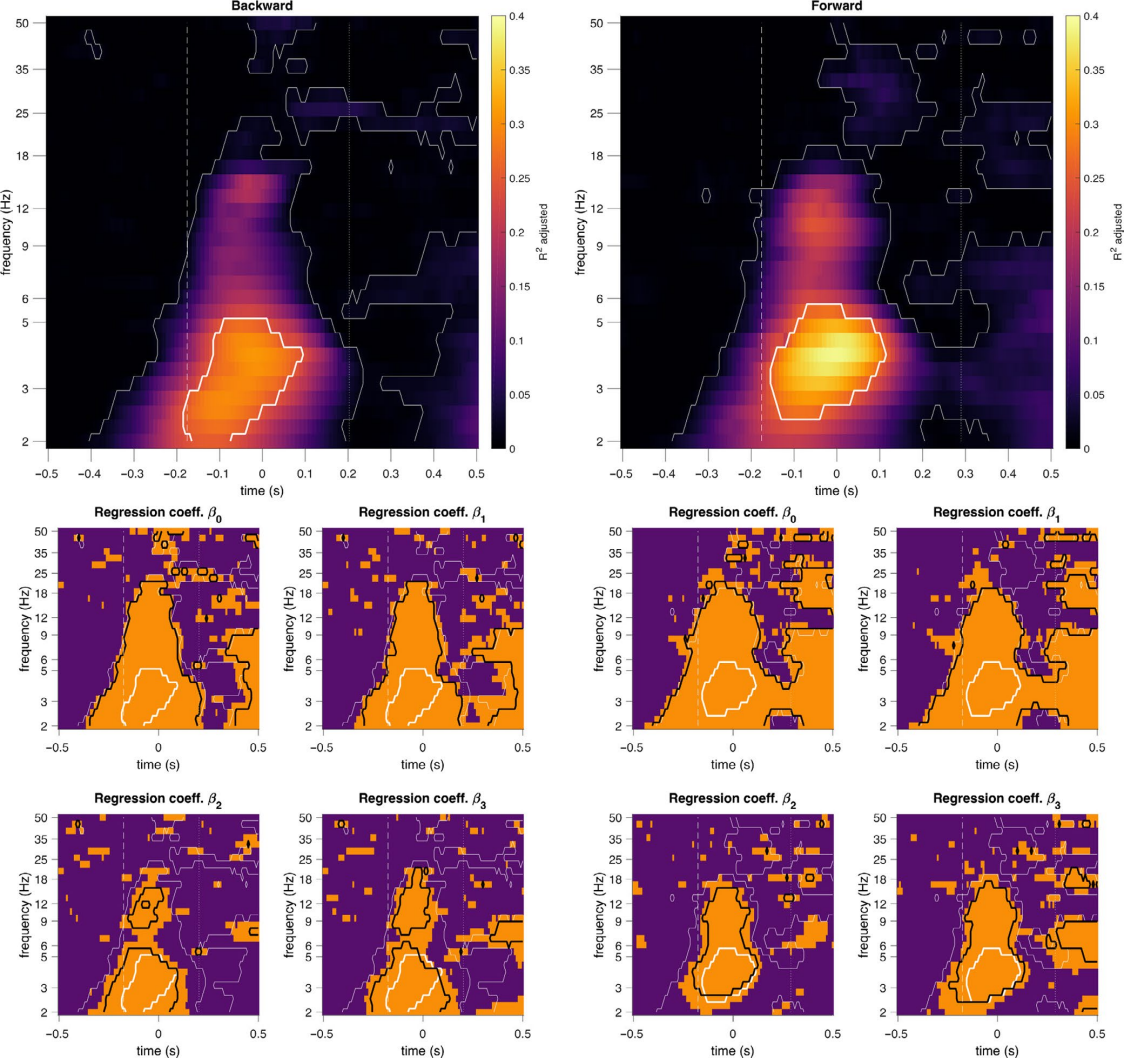
Relation of perturbation intensity and stepping responses with time‐frequency domain parameters. Time‐frequency maps of adjusted determination coefficients (top row) and regression coefficients (middle and bottom rows) shown relative to the peak amplitude of the N1 potential (*t* = 0 s). The regression coefficient β_0_ is the intercept of the model, β_1_ indicates the effect of acceleration, β_2_ indicates the effect of distinct reactive postural responses, and β_3_ indicates the effect of the interaction between acceleration and distinct reactive postural responses. The vertical dashed line indicates the median perturbation onset latency (backward and forward: −175.8 ms) and the vertical dotted line indicates the median foot‐off latency (backward: 202.6 ms; forward: 289.9 ms). A thin white contour indicates statistically significant determination coefficients, whereas a thick white contour line highlights the top 5% determination coefficients. Time‐frequency bins with non‐zero regression coefficients (estimated from the 95% confidence intervals) are shown in orange. A thick black contour indicates statistically significant regression coefficients. All significance levels are *p* < .01, FDR corrected

Figure 7 shows time‐frequency maps of the adjusted determination coefficient and the corresponding regression coefficients obtained from the regression analyses using spectral parameters. These maps show statistically significant effects of perturbation intensity, stepping behavior, and their interaction, in the time interval between perturbation onset and reactive responses and over a broad frequency band. The time interval corresponds with the expected interval of the N1 potential and the broad frequency band correspond with power increase revealed from the event‐related spectral modulations time‐frequency maps (Figure 4).

The strongest relation with spectral parameters (indicated by the highest determination coefficient) occurred at ~4 Hz and −20 ms for the backward direction (*R* ^2^ = .3079, *F*(691) = 104, *p* = 1.61e−55) and ~4 Hz and −10 ms for the forward direction (*R* ^2^ = .3895, *F*(672) = 145, *p* = 2.83e−72); with statistically significant effects of perturbation intensity (backward: *p* = 9.54e−15; forward: *p* = 2.91e−33), stepping behavior (backward: *p* = 7.49e−07; forward: *p* = 3.09e−08), and the interaction between them (backward: *p* = 5.70e−06; forward: *p* = 7.74e−11).

**FIGURE 8:**
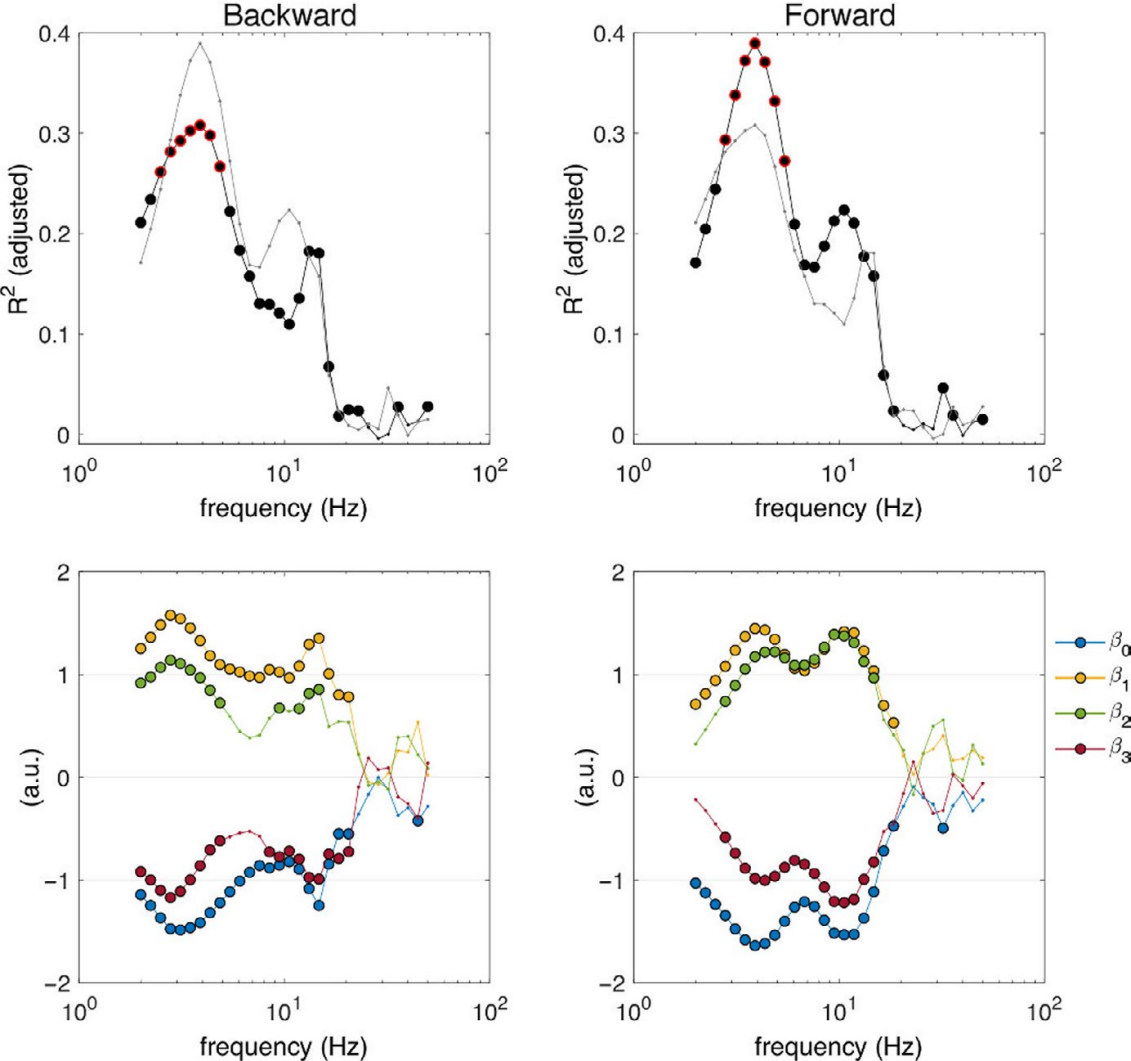
Spectral distribution of determination and regression coefficients. Frequency‐dependent adjusted determination coefficient (top) and regression coefficients (bottom) for the time point with the highest determination coefficient (backward: −20 ms; forward: −10 ms). For comparison, the determination coefficient for the opposite direction is overlaid with a gray thin line. Filled circles indicate significant coefficient values (*p* < .01, FDR corrected). The top 5% determination coefficients across all time frequency bins per direction are indicated with a red edge (top row only). The regression coefficient β_0_ is the intercept of the model, β_1_ indicates the effect of acceleration, β_2_ indicates the effect of distinct reactive postural responses, and β_3_ indicates the effect of the interaction between acceleration and distinct reactive postural responses

**FIGURE 9:**
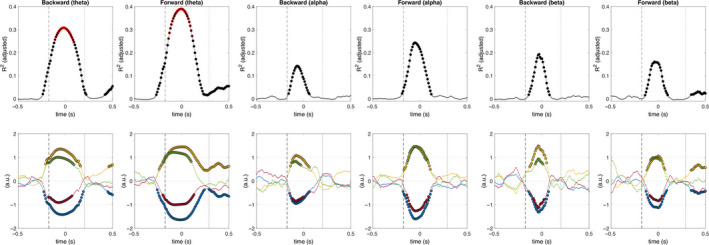
Temporal evolution of determination and regression coefficients for theta, alpha, and beta frequencies. Time‐dependent adjusted determination coefficient (top row) and regression coefficients (bottom row) shown relative to the peak amplitude of the N1 potential (*t* = 0 s). The regression coefficient β_0_ is the intercept of the model, β_1_ indicates the effect of acceleration, β_2_ indicates the effect of distinct reactive postural responses, and β_3_ indicates the effect of the interaction between acceleration and distinct reactive postural responses. The vertical dashed line indicates the median perturbation onset latency (backward and forward: −175.8 ms) and the vertical dotted line indicates the median foot‐off latency (backward: 202.6 ms; forward: 289.9 ms). Filled circles indicate significant coefficient values (*p* < .01, FDR corrected). The top 5% determination coefficients across all time‐frequency bins per direction are indicated by a red edge (top row only)

The spectral distribution of the determination coefficients at the best‐fit time points (shown in Figure 8), shows distinct peak in the determination coefficients at ~11 Hz (forward) and ~15 Hz (backward). Although a direct comparison between the two directions was not pursued, it is clear that the distribution of their determination coefficients is different, with higher determination coefficients for the forward direction and slightly different peak frequencies (indicated by the regression coefficients) between directions. The effect of perturbation intensity was statistically significant for both directions from 2 to 21 Hz (backward: *p* < .00026; forward: *p* < .0039), but the effect of stepping behavior was statistically significant between 2–5 Hz and 9–15 Hz for the backward direction (*p* < .0012) and between 3–15 Hz for the forward direction (*p* < .0011). Furthermore, peak coefficients occurred at 3 and 15 Hz (backward), and 5 and 9 Hz (forward). The interaction between perturbation intensity and stepping behavior followed a similar pattern (backward: *p* < .0015; forward: *p* < .0018). Figure 9 shows the time course of the regression analyses for 4, 11, and 15 Hz. Overall, the time course of the adjusted determination coefficients shows statistically significant effects of perturbation intensity, stepping behavior, and their interaction slightly preceding the time of the N1 potential (*t* = 0 s).

## 4 DISCUSSION

The key finding in our study is that the association between early cortical responses and the perturbation intensity differs according to the ensuing behavioral response to restore balance. This could not have been revealed in previous studies because differences in cortical responses between stepping and non‐stepping behavior were confounded by the effects of perturbation intensity (i.e., different behavioral responses were elicited by different perturbation intensities). Our analyses show that the characteristics of the N1 potential (peak power and latency) and the power of the theta, alpha, and beta rhythms index the magnitude of an imposed deviation from postural stability and the execution of late‐phase reactive postural responses, in both forward and backward perturbation directions. The peak power and latency of the N1 potential rapidly scales with increasing perturbation intensities that elicit feet‐in‐place responses (consistent with previous studies), but the scaling is attenuated when the perturbation intensities are high enough to elicit stepping responses. Additionally, our analyses show that the power of theta, alpha, and beta rhythms is similarly modulated, but that the theta rhythm has a stronger association with the interaction between perturbation intensity and the ensuing postural response than the alpha and beta rhythms. Our results indicate that scaling of cortical responses with perturbation intensity appears to be consistent with a behavioral model of stepping probability. Hence our study presents evidence that ties together the cortical responses to balance perturbations and balance recovery behavior.

### 4.1 Cortical balance control: Monitoring postural stability to predict balance recovery behavior

Our finding on the significant association of cortical responses with the interaction between perturbation intensity and the ensuing postural response (Figures 5, 6, 7, 8), provides new evidence of the possible cortical contributions to the decision‐making process for selection of appropriate postural responses. The cerebral cortex may contribute to maintaining postural stability by monitoring deviations from a stable postural state and modulating or initiating appropriate balance corrective responses. Whole‐body perturbations to standing balance elicit bouts of multisensory information (visual, vestibular, proprioceptive) that are proportional to the direction, magnitude, and rate of change in postural stability. This multisensory information is quickly integrated at subcortical levels of the central nervous system to produce very fast, yet highly coordinated, automatic postural responses (Lockhart & Ting, 2007; Welch & Ting, 2008, 2009, 2014). At the cortical level, multisensory information may be further processed to determine the need for late‐phase responses and/or an update to the current motor plan (Bolton, 2015; Jacobs & Horak, 2007; Maki & McIlroy, 2007). The need for late‐phase balance recovery responses (e.g., stepping) could be determined by comparing an ongoing change in postural stability against an internal reference of expected or acceptable deviation from a stable posture (Lockhart & Ting, 2007; Safavynia & Ting, 2013; Scott, 2012; Welch & Ting, 2014). This internal reference may be dynamically adapted (Lockhart & Ting, 2007) according to experience (e.g., predictability or habituation) and physical (e.g. biomechanical configuration, posture), environmental, and task constraints (e.g. postural threat or postural demand). Indeed, the perturbation‐induced N1 potential has previously been suggested to represent the deviation from stable posture, as a form of error detection (Adkin et al., 2006; Payne, Ting, et al., 2019), as it scales with perturbation intensity and its corresponding destabilizing effect. Furthermore, the factors that could adapt the internal reference for acceptable deviations from postural stability are known to have an impact on the amplitude of the N1 potential (see Section 1). Our results provide further evidence that support the role of the N1 potential (and associated cortical rhythms) in monitoring postural stability as a deviation from a stable postural state.

Because the N1 potential and the accompanying modulations of theta, alpha, and beta rhythms, precede the actual stepping responses by hundreds of milliseconds, we suggest that these reflect cortical processes involved in monitoring postural stability to predict the need for stepping responses. Our regression models for behavioral (Figure 2) and cortical responses (Figure 5) show that as perturbation intensity and stepping probability increase, the peak power of the N1 potential increases and its latency shortens. The experimental data shows rapid changes in peak power and latency associated with low‐intensity perturbations and near‐zero stepping probability, which are followed by modest changes in peak power and latency associated with higher‐intensity perturbations and higher stepping probability. We propose that the marked changes in peak power and latency associated with increasing perturbation intensity represent the neural computations that signal the growing need for stepping responses, which is more evident at low‐intensity perturbations before the need for stepping responses becomes certain at high‐intensity perturbations.

The mapping between cortical responses and stepping probabilities could be modulated by the experimental paradigm. The perceived growing need for stepping responses may be modulated by the uncertainty regarding perturbation onset, intensity, and direction, and the relatively high probability of high‐intensity perturbations that require stepping responses. When uncertainty about postural demand or postural threat exists, the central nervous system is conservatively driven toward a default state in anticipation of high postural demand/threat (Mochizuki et al., 2010). Thus, the experimental conditions and task instructions could set the relation between stepping probability and cortical responses. To further validate the role of the cerebral cortex in the prediction of stepping responses it will be necessary to manipulate the internal reference for postural stability, perhaps by altering the distribution of perturbation intensities (e.g., including catch trials) or directly manipulating postural stability. Manipulating postural stability could be achieved, for instance, by controlled displacement of the center of mass relative to the base of support, prior to the onset of a balance perturbation.

Although our results show an association between cortical responses and the ensuing balance recovery behavior, further analyses are necessary to uncover any causal effect of cortical and postural responses (see Peterson and Ferris (2019)). Future studies must pursue time‐dependent analyses of (effective) cortico‐muscular connectivity to better understand the top‐down regulation of balance recovery responses.

### 4.2 Estimated cortical source and interpretation of the N1 potential

We estimated the cortical source of the N1 potential in the posterior midline frontal cortex (Table 3; Figure 4), near the SMA. Although the spatial resolution of our source localization analysis is limited by the use of standard electrode positions and head model, the estimated cortical source is consistent with previous studies on cortical involvement in balance control (Marlin et al., 2014; Mierau et al., 2015). The localization of the N1 potential to the SMA has been considered as an indication that the N1 potential may be related to sensorimotor processes (e.g., movement preparation and initiation) instead of mechanisms of cognitive control (Marlin et al., 2014; Mierau et al., 2015; Solis‐Escalante et al., 2019; Varghese et al., 2017). However, different aspects of cognitive control are associated with error‐related potentials ERN/ErrP and modulations of the theta rhythm from the SMA and other structures in the posterior midfrontal cortex. The SMA has been long implicated in action monitoring and adaptive behavior (Bonini et al., 2014; Cohen, 2014; Luu et al., 2000; Ridderinkhof et al., 2004). Error‐related potentials and modulations of theta rhythm in or near the SMA signal the need for corrective actions (Pereira et al., 2017; Töllner et al., 2017). In our study, the presumed cortical source of the N1 potential provides further evidence for its involvement in cognitive control, for example, in action monitoring.

### 4.3 Different rhythms and distinct aspects of the control of balance and posture

Our results show that, in addition to the modulations in the midfrontal theta rhythm discussed above, the power of the alpha and beta rhythms was also related to perturbation intensity and balance recovery behavior (Figures 7 and 8). Because different rhythms have been associated with distinct cognitive and sensorimotor functions (Buzsaki, 2006), it is plausible that the theta, alpha, and beta rhythms represent distinct aspects of the cortical control of balance. The midfrontal theta rhythm is a known marker of cognitive control and action monitoring (Cavanagh & Frank, 2014; Cohen & Donner, 2013), whereas the alpha and beta rhythms are classical sensorimotor rhythms with cortical sources over bilateral sensorimotor cortices (Pfurtscheller & Lopes da Silva, 1999). Since it has been shown that the SMA acts as a hub for information flow related to sensorimotor and cognitive processes elicited by external perturbations (Peterson & Ferris, 2019), it is possible that the alpha and beta rhythms observed in our analyses are related to communication between the SMA and the sensorimotor cortices. With respect to balance control, power modulations of alpha and beta rhythms have been reported near the bilateral M1/S1 and the midfrontal SMA during the preparation and execution of balance recovery responses with feet‐in‐place and stepping responses (Peterson & Ferris, 2018; Solis‐Escalante et al., 2019). Future studies should consider frequency‐specific analyses to disentangle the functional role of individual cortical rhythms.

### 4.4 Relevance of perturbation direction

Although we did not formally evaluate the effect of perturbation direction on the cortical responses, it is worth mentioning that we found higher determination coefficients for perturbations that elicit postural sway in the forward direction (Figures 6 and 8), and distinct spectral distributions of the determination and regression coefficients between perturbation directions (Figure 8). Moreover, our behavioral analyses (Figure 2; Table 1) showed differences in the proportions of stepping trials and mean stepping latencies of the two perturbation directions. These results suggest the existence of functional differences in the cortical responses to distinct perturbations that may be of interest for future studies.

Previous studies have found that the amplitude of the N1 potential is not modulated by the direction of the perturbation (Dietz, Quintern, Berger, et al., 1985; Goel et al., 2018; Payne, Hajcak, et al., 2019). However, reactive postural responses are direction‐specific (Chvatal et al., 2011; de Kam et al., 2018; Torres‐Oviedo & Ting, 2007), and therefore, cortical processes involved in top‐down control of balance and posture could carry direction‐specific information. Future studies may focus on finding direction‐specific modulations of the multiple cortical rhythms that accompany the N1 potential.

### 4.5 Limitations

In our experiment, we controlled the distribution of the balance perturbations to maintain the unpredictability of perturbation intensity and perturbation direction. The near‐uniform distribution of the perturbation intensities led to different proportions of feet‐in‐place and stepping responses in the two stepping directions, with a slight bias toward stepping responses in the backward (63%) and forward (53%) stepping directions. It could be argued that the effect of stepping behavior is overestimated in the regression models for cortical responses. However, the visualization of the data (Figure 5) suggests that the effect of stepping behavior is primarily driven by the responses to low‐intensity perturbations and feet‐in‐place responses. Moreover, the significantly different proportions of stepping trials comparing the backward and forward stepping directions led to qualitatively similar results, suggesting that an effect of sampling bias is negligible.

## 5 CONCLUSIONS

Our study expands the understanding of cortical contributions to balance control by demonstrating that the N1 potential, theta (~4 Hz), alpha (~11 Hz), and beta (~15 Hz) rhythms, arising from the midfrontal cortex, index the magnitude of a sudden deviation from postural stability during quiet stance and appear to be involved in the prediction of eventual stepping responses. The relation of the cortical responses to whole‐body balance perturbations with the intensity of the perturbation (as a form of perceived error), and the ensuing reactive postural response (as necessary corrective actions), provide further evidence that cognitive control mechanisms (e.g., action monitoring) may regulate reactive postural adjustments for maintaining postural stability.

## CONFLICT OF INTERESTS

None.

## AUTHOR CONTRIBUTIONS

TSE, VW, MXC conceived and designed research; TSE performed experiments; TSE, MXC contributed analysis tools; TSE analyzed data; TSE, VW, MXC, MS interpreted results of experiments; TSE drafted manuscript; TSE, MS, VW, MXC edited and revised manuscript. All authors approved the final version of manuscript.

### PEER REVIEW

The peer review history for this article is available at https://publons.com/publon/10.1111/ejn.14972.

## Supporting information

Fig S1

## Data Availability

Data used in this article is available from the authors upon reasonable request.
