# Supplementary material for: Cortical responses to whole‐body balance perturbations index perturbation magnitude and predict reactive stepping behavior
Source: Eur J Neurosci. 2020 Sep 20;54(12):8120–38. doi: 10.1111/ejn.14972 (PMC9290492; doi:10.1111/ejn.14972)
Supplement: Supplementary file 1 — Fig S1 [file EJN-54-8120-s001.pdf]

Relation of spectral parameters with perturbation intensity and stepping behavior.

Figure A shows the pooled single-trial power of the theta, alpha, and beta rhythms, together with their corresponding regression models. The data corresponds to the time-frequency bins that yielded the highest (adjusted) determination coefficients. All determination and regression coefficients indicated significant effects ($p < 0.01$) of perturbation intensity, stepping behavior, and the interaction between these factors. These results show that the theta, alpha, and beta rhythm follow (to a certain degree) the characteristic of the N1 peak power, namely, a rapid increase with perturbation intensity in trials with non-stepping responses, followed by an less marked increase with perturbation intensity in trials with stepping responses. The determination coefficients and single-trial power show that the model is better adjusted to the power of the theta rhythm.

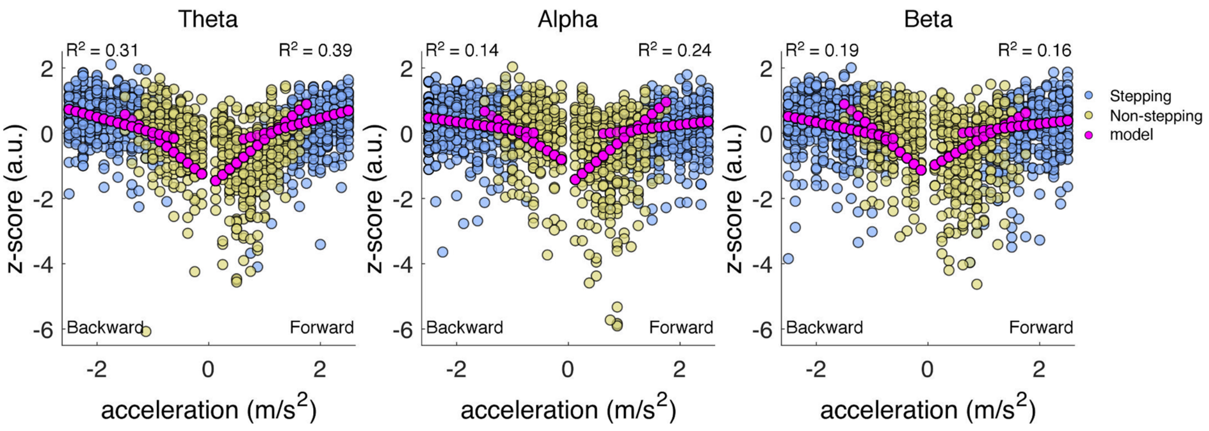

Figure A. Relation of perturbation intensity and stepping responses with single-trial power of the theta, alpha, and beta rhythms. Normalized power of each individual rhythm with respect to perturbation intensity (i.e., acceleration magnitude). The x-axis indicates perturbation intensity multiplied by the sign of the sway direction (negative: backward sway; positive: forward sway). Blue circles indicate trials with a stepping response and yellow circles indicate trials with a non-stepping response. The corresponding regression models are shown with magenta circles. The relation with stepping behavior is indicated by the change in slope of the regression models seen between trials with stepping and non-stepping responses. In our analyses, the time-frequency bins for highest determination coefficients resulted in frequencies: theta 3.89 Hz; alpha 10.57 Hz; beta 14.75 Hz; and latencies: theta backward −20 ms, theta forward −10 ms; alpha backward −70 ms, alpha forward −50 ms; beta backward −30 ms, theta forward −40 ms.
